# Difference in the prevalence of hypertension when measured according to the American Heart Association and the European Society of Cardiology hypertension cut-offs in the ELSA-Brasil cohort

**DOI:** 10.1590/1414-431X2025e14320

**Published:** 2025-11-14

**Authors:** A.L.F. Favaretto, B.B. Duncan, M.I. Schmidt, M.S. Bittencourt, S.M. Barreto, A.B.S. Santos, M. Foppa, L.B. Moreira

**Affiliations:** 1 Programa de Pós-Graduação em Cardiologia, Universidade Federal do Rio Grande do Sul, Porto Alegre, RS, Brasil; 2 Programa de Pós-Graduação em Epidemiologia, Faculdade de Medicina, Universidade Federal do Rio Grande do Sul, Porto Alegre, RS, Brasil; 3 Department of Internal Medicine and Department of Radiology, University of Pittsburgh School of Medicine, Department of Internal Medicine and Department of RadiologyUniversity of Pittsburgh School of MedicineUSAUSA; 4 Departamento de Medicina Preventiva e Social, Universidade Federal de Minas Gerais, Belo Horizonte, MG, Brasil; 5 Departamento de Cardiologia, Hospital de Clínicas de Porto Alegre, Programa de Pós-Graduação em Cardiologia, Universidade Federal do Rio Grande do Sul, Porto Alegre, RS, Brasil; 6 Programa de Pós-Graduação em Cardiologia, Hospital de Clínicas de Porto Alegre, Universidade Federal do Rio Grande do Sul, Porto Alegre, RS, Brasil

**Keywords:** Hypertension, Blood pressure, Diagnosis, Cardiovascular risk, Prevalence

## Abstract

The 2017 US guidelines for the prevention, detection, evaluation, and management of high blood pressure in adults proposed the diagnosis of hypertension at 130/80 mmHg, while the European Society of Cardiology and 2020 Brazilian Guidelines of Hypertension maintain the 140/90 mmHg cut-off. We aimed to evaluate how the cut-off established by the American Heart Association guidelines would impact the prevalence of hypertension in the ELSA-Brasil cohort and compare the clinical characteristics among these subgroups. The participants were part of the ongoing ELSA-Brasil multicenter cohort, with baseline data collected between 2008 and 2010, consisting of 15,105 public servants of both sexes aged 35 to 74 years. Hypertension (≥140 or ≥90 mmHg or use of antihypertensive drugs in the last two weeks if below these values) prevalence was 36.2% (95%CI: 35.4-36.9, n=5,456) with the Brazilian cut-off and 51.4% (95%CI: 50.6-52.1, n=7,756) when considering the US cut-off (SBP≥130 or DBP≥80 mmHg). In general, those with high blood pressure (HBP) presented an intermediate-risk profile compared to the hypertension group. Lowering the hypertension cut-off caused an absolute increase of 15.2% in the prevalence of hypertension in the sample of public servants studied. HBP individuals showed intermediate-risk profile between normal blood pressure and hypertension and represented a large fraction of the population who may benefit from treatment.

## Introduction

Hypertension (HT) is the most important modifiable risk factor for cardiovascular events. Every 20 mmHg of systolic blood pressure (SBP) increase or 10 mmHg of diastolic blood pressure (DBP) increase doubles the risk (1). SBP>115 or DBP>75 mmHg explains 49% of the incidence of coronary events and 68% of strokes (2). However, whether people with these values would benefit from treatment for coronary heart disease is controversial. As lower blood pressure (BP) values are known to be beneficial, the American Heart Association guidelines (3) for the prevention, detection, evaluation, and management of high blood pressure in adults has established a new threshold for the diagnosis of HT of 130/80 mmHg. In comparison, both the European Society of Cardiology (4) and Brazilian (5) guidelines maintain 140/90 mmHg as the cut-off to diagnose hypertension (6), recommending a therapeutic target of <120/80 mmHg. Considering the divergences regarding the threshold for HT diagnosis, this study aimed to evaluate the impact of including those with high blood pressure (HBP: SBP≥130 or DBP≥80 mmHg) on the prevalence of HT in the ELSA-Brasil cohort. Additionally, we compared demographic data, prevalence of cardiovascular risk factors, comorbidities, and HT control rate between patients with hypertension as defined by the two cut-off values.

## Material and Methods

The participants were part of the ELSA-Brasil study, an ongoing Brazilian multicenter cohort with baseline data collected between 2008 and 2010, consisting of 15,105 public servants of both sexes aged 35 to 74 years from universities and research institutions in six Brazilian capitals (7). The ELSA-Brasil study was approved by each investigation center's institutional ethics committee, and written informed consent was obtained from all participants.

Study participants were evaluated in the research clinic at study baseline following specific protocols (8). SBP and DBP were measured in triplicate at 1-min intervals after 5 min at rest, sitting without crossed legs, in a quiet environment, and with temperature ranging from 20 to 24°C. The device used was Omron HE M 705CPINT (Japan). The average of the last two measures were considered. BP was categorized according to normal BP (SBP<130 and DBP<80 mmHg), high BP (SBP≥130 to <140 mmHg and/or DBP≥80 to <90 mmHg - US guideline), and hypertension (SBP≥140 or DBP≥90 mmHg - European/Brazilian guidelines). Participants taking antihypertensive drugs were categorized as hypertensive.

Socioeconomic, demographic, anthropometric, clinical, and lifestyle information were obtained with questionnaires and clinical examination following standardized protocols. Annual per capita family income was recorded in Brazilian reals and converted to US dollars (USD) using the exchange rate of June 30, 2009 (BR$ 1.95=US$ 1.00). Excessive alcohol consumption was classified as alcohol intake above 210 g/week for men and 140 g/week for women. Abdominal obesity was considered as an abdominal circumference above 102 cm for men and 88 cm for women. Leisure-time physical activity and commuting to work were classified as intense, moderate, and weak considering minutes per week of physical activity (9).

Prevalent co-morbidities were self-reported and complemented by electrocardiography and the brachiotibial index. Peripheral arterial disease was confirmed by self-report of intermittent claudication, a brachiotibial index below 0.90, or both. Prevalent cardiovascular disease was characterized by a history of stroke, heart failure, acute myocardial infarction, cardiac revascularization, or the presence of electrocardiographic alterations at baseline. Severe coronary disease included a history of acute myocardial infarction, cardiac revascularization, or the presence of electrocardiographic alterations. The cardiovascular risk for 10 years was calculated using the American College of Cardiology (ACC)/American Heart Association (AHA) atherosclerotic cardiovascular disease (ASCVD) risk estimator (10). More details have been described in previous publications (7,8,11).

The statistical analysis was performed using the software R Studio version 4.2.1. We applied descriptive statistics, chi-squared, and ANOVA or Kruskal-Wallis tests to compare the characteristics between the three groups. The normal BP group comprised the BP <130/80 mmHg; high-normal BP, from 130/80 to <140/90 mmHg, and hypertension, ≥140/90 mmHg or use of antihypertensive drugs. Additional analyses stratified by age groups were performed. The prevalence of HT according to the BP cut-off points was described with a 95%CI. Differences with P-values <0.05 were considered statistically significant. The sample size of 15,087 participants had 95% power to detect a difference of 5% between the two classifications based on the cut-off for hypertension (12).

## Results

Of the 15,105 participants, 18 did not have BP recorded. We included in the analysis 15,087 public servants. Overall sociodemographic data are presented in Table 1. The sample had slightly more women, 54.4% (n=8,211), the mean age of 52 years (±9.1), and the median annual per capita family income in US dollars (USD) of $2,634 (ranging from $1,416 and $4,448). The majority, 52.6% (n=7,941), had higher education and 34.6% (n=5,229) had completed high school. Most participants' skin color/race was white (52.2%, n=7,785), blacks and mixed race 43.7%, Asian 2.5%, and indigenous 1.1%. Mean SBP of the sample was 121.3 mmHg (±17.3) and DBP 76.3 mmHg (±10.8).

**Table 1:** Sociodemographic characteristics overall, by blood pressure category, and by blood pressure cut-off.

Variable	Sample (n=15,087)	SBP<130 and DBP<80 (n=7331)	SBP≥130-<140 or DBP≥80-<90 (n=2300)		SBP≥140 or DBP≥90 (n=5456)	P	SBP≥130 or DBP≥80 (n=7756)
Male	6876 (45.6)	2769 (37.8)	1326 (57.7)		2781 (51)	<0.001	4107 (53)
Age (years)	52 (±9.1)	49.5 (±8.5)	50.9 (±8.3)		56.0 (±8.8)	<0.001	54.5 (±9.0)
Age group						<0.001	
35 to 44 years old	3338 (22.1)	2275 (31)	531 (23.1)		532 (9.8)		1063 (13.7)
45 to 54 years old	5934 (39.3)	3028 (41.3)	1029 (44.7)		1877 (34.4)		2906 (37.5)
55 to 64 years old	4227 (28.0)	1623 (22.1)	588 (25.6)		2016 (37)		2604 (33.6)
65 to 74 years old	1588 (10.5)	405 (5.5)	152 (6.6)		1031 (18.9)		1183 (15.2)
Schooling						<0.001	
Incomplete elementary	891 (5.9)	250 (3.4)	130 (5.7)		511 (9.4)		641 (8.3)
Complete elementary	1026 (6.8)	354 (4.8)	160 (7.0)		512 (9.4)		672 (8.7)
High school	5229 (34.6)	2370 (32.3)	837 (36.4)		2022 (37.1)		2859 (36.9)
College	7941 (52.6)	4357 (59.4)	1173 (51.0)		2411 (44.2)		3584 (46.2)
Skin color	14921					<0.001	
Black	2397 (15.9)	870 (11.9)	358 (15.6)		1169 (21.4)		1527 (19.7)
Mixed	4200 (27.8)	1925 (16.3)	697 (30.3)		1578 (28.9)		2275 (29.3)
White	7785 (51.6)	4201 (57.3)	1136 (49.4)		2448 (44.9)		3584 (46.2)
Other	531 (3.5)	265 (3.6)	79 (3.4)		187 (3.4)		266 (3.4)

Data are reported as n (%) or mean (SD). Chi-squared test and *t*-test. SBP: systolic blood pressure; DBP: diastolic blood pressure.

HT, when defined as SBP≥140 or DBP≥90 mmHg or use of antihypertensive drugs in the last 2 weeks, had a prevalence of 36.2% (95%CI: 35.4-36.9, n=5,456). Considering SBP≥130 or DBP≥80 mmHg according to the US guideline, the prevalence rose to 51.4% (95%CI: 50.6-52.1, n=7,756). Of all participants, 27.8% (n=4,203) had used antihypertensive drugs in the last two weeks. Of these, 40.8% had BP<130/80 mmHg (n=1,714), 29.1% were in the high BP category (n=1,224), and 30.1% remained in the HT range (n=1,265).

Participant characteristics categorized according to normal BP (SBP<130 and DBP<80 mmHg), high BP (SBP≥130 to <140 mmHg and/or DBP≥80 to <90 mmHg), and HT are presented in Table 1. The groups had mean SBP of 111 (±9.3), 128 (±7.2), and 149 mmHg (±14.6), respectively, and mean DBP of 69.7 (±6.2), 82.3 (±4.6), and 91.4 mmHg (±9.5), respectively. In the HT group, the prevalence of men was 51%, while in the high BP group it was 57.7%. The mean age (56 ±8.8 years) was higher in the HT group than in the other categories, the educational attainment was lower, and the prevalence of self-declared black skin color/race was greater (21.4%).

**Figure 1:**
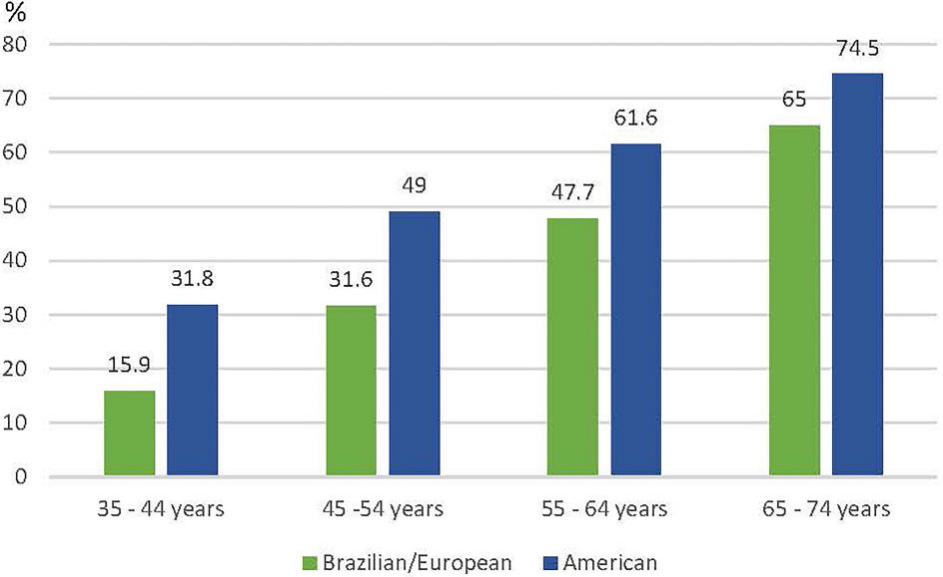
Prevalence of hypertension by age group and blood pressure criteria (%). Brazilian/European guideline: Hypertension - SBP≥140 or DBP≥90mmHg; American guideline: Hypertension - SBP≥130 or DBP≥80mmHg.

Figure 1 shows that the prevalence of HT increased with age when using both criteria; however, the prevalence of HT was higher when using the US criteria in each age group. The age group from 55 to 64 years had the highest prevalence (37%) when HT was defined according to the European and Brazilian guidelines. The range between 45 and 54 years had the highest prevalence (37.5%) when HT was defined according to the current US guideline Table 1.

Table 2 describes the cardiovascular risk factors. Weight, waist-hip ratio, and body mass index were significantly higher in the HT group. The prevalence of the other risk factors and cardiovascular comorbidities was also higher in the HT group (P for trend <0.001) (Table 3).

**Table 2:** Cardiovascular risk factors overall and by blood pressure category.

Variable	Sample (n=15087)	SBP<130 and DBP<80 (n=7331)	SBP≥130-<140 or DBP≥80-<90 (n=2300)	SBP≥140 or DBP≥90 (n=5456)	P
Weight (kg)	73.7 (±15.1)	69.5 (±13.1)	76.6 (±15.1)	78.1 (±16.1)	<0.001
Waist-hip ratio	0.896 (±0.09)	0.86 (±0.08)	0.91 (±0.08)	0.93 (±0.08)	<0.001
BMI (kg/m^2^)	27.0 (±4.74)	25.6 (±4.05)	27.5 (±4.72)	28.7 (±5.01)	<0.001
Abdominal obesity					<0.001
No	9666 (64.1)	5538 (75.5)	1443 (62.7)	2685 (49.2)	
Yes	5418 (35.9)	1793 (24.5)	857 (37.3)	2768 (50.7)	
Alcohol use					<0.001
Never used	1614 (10.7)	739 (10.1)	225 (9.8)	650 (11.9)	
Former	3034 (20.1)	1384 (18.9)	475 (20.7)	1175 (21.5)	
User	10422 (69.2)	5201 (70.9)	1599 (69.5)	3622 (66.4)	
Excessive drinker^a^	1123 (7.4)	387 (5.3)	203 (8.8)	533 (9.8)	<0.001
Smoking habit					<0.001
Never smoked	8585 (56.9)	4390 (59.9)	1311 (57.0)	2884 (52.9)	
Former smoker	4525 (30.0)	1922 (26.2)	688 (29.9)	1915 (15.1)	
Smoker	1976 (13.1)	1019 (13.9)	301 (13.1)	656 (12.0)	
Physical activity					<0.001
Intense	1047 (6.9)	595 (8.1)	164 (7.1)	288 (5.3)	
Moderate	2395 (15.9)	1163 (15.9)	368 (16.0)	864 (15.8)	
Weak	11432 (75.8)	5452 (74.4)	1734 (75.4)	4246 (77.8)	
Diabetes Mellitus	2424 (16.1)	523 (7.1)	300 (13.0)	1601 (29.3)	<0.001
Coronary heart disease in the family (age <60)	2314 (15.3)	998 (13.6)	334 (14.5)	982 (18.0)	<0.001

Data are reported as n (%) or mean (SD). Chi-squared test and *t*-test. BMI: body mass index; SBP: systolic blood pressure; DBP: diastolic blood pressure. ^a^Excessive drinker: Men ≥210 g alcohol/week; women ≥140 g alcohol/week.

**Table 3:** Prevalence of comorbidities in the sample by blood pressure category.

Comorbidities	Events (n=15087)	SBP<130 and DBP<80 (n=7331)	SBP≥130-<140 or DBP≥80-<90 (n=2300)	SBP≥140 or DBP≥90 (n=5456)	P
Peripheral arterial disease	656 (4.3)	226 (3.1)	80 (3.5)	350 (6.4)	<0.001
Cardiovascular disease	999 (6.6)	237 (3.2)	93 (4.0)	669 (12.3)	<0.001
Severe coronary disease	678 (4.5)	162 (2.2)	68 (3.0)	448 (8.2)	<0.001

Data are reported as n (%). Chi-squared test and *t*-test. SBP: systolic blood pressure; DBP: diastolic blood pressure.

**Figure 2:**
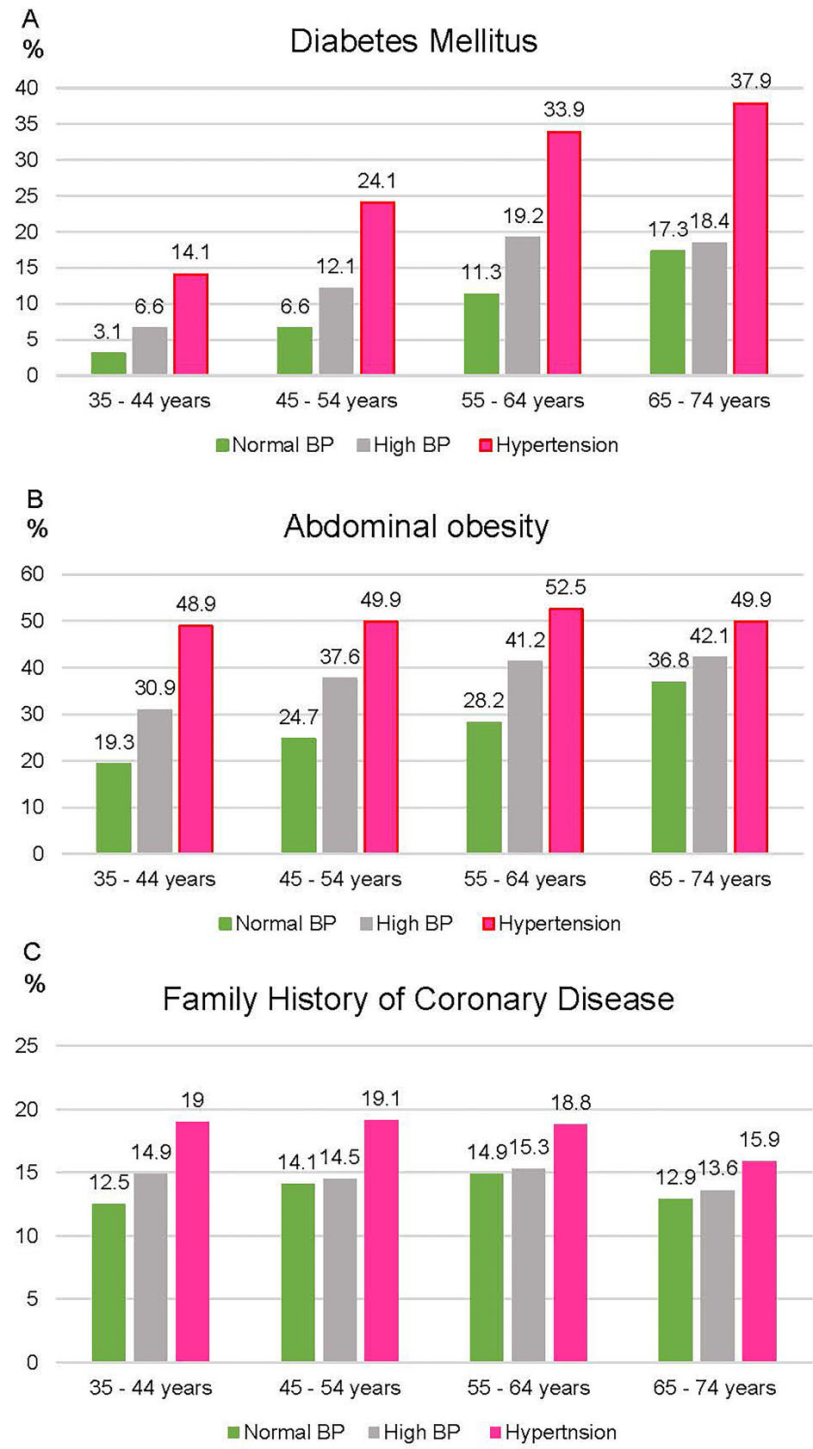
Prevalence of diabetes mellitus (**A**), abdominal obesity (**B**), and family history of coronary disease (**C**) by age group and blood pressure category (%). SBP: systolic blood pressure; DBP: diastolic blood pressure.

The prevalence of diabetes mellitus (DM) in the sample increased with age, rising from 5.4% in the 35 to 44 years to 30% in the 65 to 74 years range. Within age groups, the prevalence of DM (Figure 2A), abdominal obesity (Figure 2B), and weight increased in absolute numbers as the BP category increased (P<0.001). Family history of coronary disease before 60 years of age was more frequent in the HT group for all age groups (Figure 2C).

**Figure 3:**
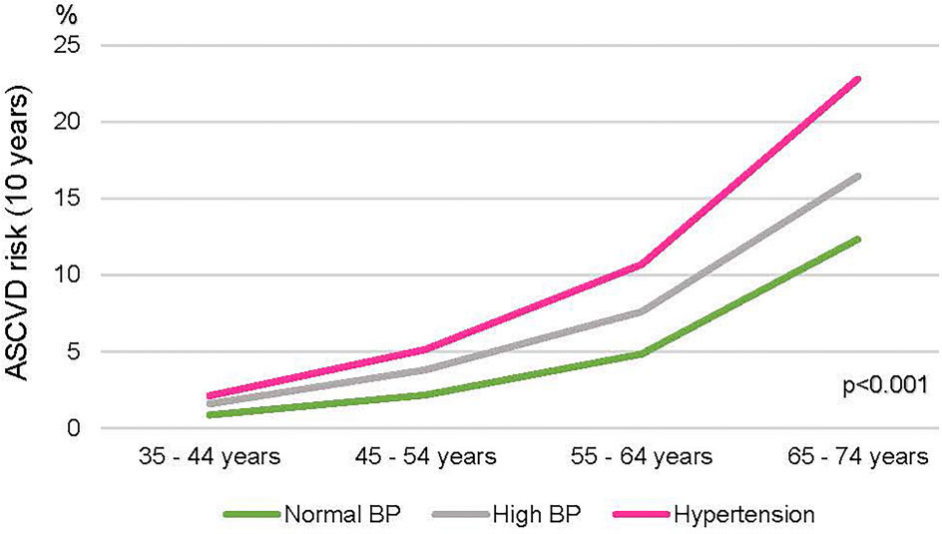
Mean atherosclerotic cardiovascular disease (ASCVD) (American Heart Association) risk in 10 years by age group and blood pressure category. BP: blood pressure.

The median ASCVD risk was higher in the HT category (normal BP, 1.45 (IQR=0.6-3.46; high BP, 3.11 IQR=1.39-6.66; HT, 6.61 IQR=2.94-13.7; P<0.001), and the elevation with increasing age was more pronounced compared to the high BP category (Figure 3).

## Discussion

The HT prevalence in the studied sample differs from the 32.5% (95%CI: 31.7-33.0), considering BP≥140/90 mmHg or use of antihypertensive drugs described by Malta et al. (13) in a nationally representative sample of Brazilian adults (18 years or more). The lower mean age of the sample in the national survey probably explains the lower rate. Compared to other countries with a prevalence of HT between 26.2 and 46.0%, our sample is in the intermediate range (14).

The number of people with HT is rising in the world and the rates of control varies between countries from 20 to 50% (15). The reduction of the BP threshold for the diagnosis of HT produced an increase of 15.2% in the prevalence of HT, compatible with that estimated by Bundy et al. (16) when comparing the differences between the 2014 and 2017 U.S. guidelines. Another study (17) recorded an increase of 13.9% (95%CI: 13.5-14.3) in the prevalence considering the same criteria. The proportion of hypertensive participants with uncontrolled BP despite using medication doubled (59.2%) when considering HT as SBP≥130 or DBP≥80 mmHg. A study that investigated the impact of the new 2017 ACC/AHA guideline from data from the NHANES 2017-2018 describes similar results (17). The 7th American guideline (18) and the 2020 Brazilian guideline (5) recommended only lifestyle changes for high BP while, by the new US criterion, those in this category should receive pharmacological treatment to reduce blood pressure for the prevention of cardiovascular diseases. Reaching the BP target to reduce cardiovascular risk is challenging.

The prevalence of risk factors and comorbidities in the group of participants with high BP is generally between that of individuals with normal BP and that of those with hypertension. It reflects, in part, the timeline of HT and cardiovascular disease development. The 45 to 55 years age range had the highest addition of hypertensive patients. The increase in the prevalence of DM and ASCVD risk according to BP in each age group does not seem to be due only to the effect of age on BP values and other risk factors. History of coronary heart disease before 60 years of age is more frequent in the HT category, in all age groups, and shows a plateau at 64 years of age. The decrease observed in the age group above 65 years is probably explained by survival bias. Abdominal obesity has a prevalence of around 50% among hypertensive participants in all age groups and rises in those with high BP up to the 55-64 age group, reaching a plateau in the group of 65-74 years. Therefore, high BP and HT groups show different profiles.

The ASCVD risk score was higher in the HT category, and the increase with increasing age was more pronounced than in the high BP category. The higher prevalence of DM in the group with high BP than in normotensives in the 35-45 years category agrees with a different pattern of cardiovascular risk. Together, the data suggest that individuals with high BP differ from both hypertensive and normal BP patients, regardless of age and beyond blood pressure. A follow-up of the cohort will allow the comparison of the incidence of cardiovascular events between the groups.

Huang et al. (19), in a meta-analysis with 19 cohort studies conducted in several countries totaling 468,561 individuals, estimated a risk ratio of 1.55 (95%CI: 1.41-1.71) for cardiovascular morbidity in prehypertensive patients (equivalent to high BP) compared to individuals with normal BP. In another meta-analysis (20) with 27 studies and approximately 500,000 participants (Asia, United States, and Europe), the risk ratio for prehypertensive patients was 1.40 (95%CI: 1.34-1.46). These data, in addition to the benefits observed in the SPRINT study (21) with reduction of the BP treatment target to values below 130/80 mmHg, support the adoption of lower cut-off points for HT diagnosis. This change is likely to lead to a growing demand for health services in primary care to treat HT and intensify the early control of cardiovascular risk factors in these individuals.

According to the 2024 European guideline (4), the diagnostic criteria for HT remains BP≥140/90 mmHg, with the addition of the elevated blood pressure category when SBP is between 120-139 mmHg or DBP is between 70-89 mmHg. Special attention is recommended during the monitoring of these individuals, especially those with moderate to severe risk of chronic kidney disease, CVD, hypertension-mediated organ damage, DM, and familial hypercholesterolemia, given their potential to become hypertensive over time and the increased 10-year risk of CVD regardless of age. Thus, the increased risk is recognized, as observed in our study, considering BP≥130/80 mmHg as part of the elevated blood pressure classification used by the European guidelines.

One limitation of our study merits discussion. While the sample was not representative of the adults from the general Brazilian population, it included a wide range of ethnicities, income, educational attainment, lifestyle habits, and adiposity measures common in Brazilian adults. Although ELSA-Brasil's sampling strategy influenced prevalence estimates, it did not invalidate the comparison of the characteristics between high BP and HT groups. Additionally, the study presents strong points: it was prospectively planned, multicenter, and with standardized data collection, which controls for potential measurement bias.

In conclusion, lowering hypertension cut-offs to SBP≥130 mmHg or DBP≥80 mmHg caused an absolute increase of 15.2% in the prevalence of HT in this sample of public servants. Regarding cardiovascular risk factors and comorbidities, participants with SBP≥130 to <140 mmHg or DBP≥80 to <90 mmHg are midway between individuals with normal BP and those with HT, suggesting that they are a distinct population, with an intermediate-risk profile. They represent a large fraction of the population who can benefit from treatment. As this group is such a large fraction of the middle-aged and elderly population, their treatment could lead to a major benefit, considering the population as a whole.

## Data Availability

All data generated or analyzed during this study are included in this published article.
